# Topographical Analysis of the Choriocapillaris Reperfusion After Loading Anti-VEGF Therapy in Neovascular AMD

**DOI:** 10.1167/tvst.11.9.18

**Published:** 2022-09-22

**Authors:** Pasquale Viggiano, Maria Oliva Grassi, Mariagrazia Pignataro, Giacomo Boscia, Enrico Borrelli, Teresa Molfetta, Federica Evangelista, Giovanni Alessio, Francesco Boscia

**Affiliations:** 1 Department of Basic Medical Sciences, Neuroscience and Sense Organs, University of Bari “Aldo Moro”, Bari, Italy; 2 Ophthalmology Department, San Raffaele University Hospital, Milan, Italy; 3 Ophthalmology Unit, A.O.U. City of Health and Science of Turin, Department of Surgical Sciences, University of Turin, Turin, Italy; 4 Ophthalmology Clinic, Department of Medicine and Science of Ageing, University G. D'Annunzio Chieti- Pescara, Chieti, Italy

**Keywords:** age-related macular degeneration (AMD), neovascular age-related macular degeneration (nAMD), optical coherence tomography (OCT), retinal disease

## Abstract

**Purpose:**

The purpose of this study was to evaluate choriocapillaris vascular density changes around macular neovascularization (MNV) before and after anti-vascular endothelium growth factor (VEGF) injections by optical coherence tomography angiography (OCTA).

**Methods:**

Treatment-naïve eyes with a diagnosis of exudative AMD and type 1 MNV were included. En face optical coherence tomography angiograms were analyzed for percentage of choriocapillaris (CC) flow deficit percentage (FD%), the FD average area (FDa), and the FD number (FDn) in 5 progressive 200-µm-wide concentric rings (R1, R2, R3, R4, and R5) surrounding the dark halo around the MNV. The OCTA acquisition was performed at the following visits: (i) before the loading phase of intravitreal injection of aflibercept or ranibizumab (T1), and (ii) 1 month after the last intravitreal injection of loading phase comprising 3 monthly injections (T2).

**Results:**

A total of 30 eyes of 30 Caucasian patients with treatment naïve neurovascular AMD (nAMD) were included in the study. All rings showed a progressive FD% reduction at T2 in comparison to T1 values indicating gradual CC reperfusion of the peripheral rings. Furthermore, we found a progressive contraction of the FD average area in all the rings considered (*P* < 0.05). On the other hand, at T2, a significant increase in the FD number of the 5 rings was displayed, as compared to T1 (*P* < 0.05).

**Conclusions:**

Our analysis showed topographical CC reperfusion after loading anti-VEGF therapy. CC flow deficits were greater around the associated dark halo before treatment, followed by a progressive recovery of CC flow after intravitreal therapy.

**Translational Relevance:**

OCTA may be used to assess the development and progression of MNV but also in assessing response to intravitreal injections of anti-VEGF.

## Introduction

Age-related macular degeneration (AMD) is a relevant cause of blindness, affecting over 6 million people globally.^1^ The development of macular neovascularization (MNV) secondary to AMD seems to be characterized by spatial distribution impairment of the choriocapillaris (CC) in the area surrounding the MNV, followed by the growth of abnormal blood vessels in the subretinal and sub-retinal pigment epithelium (RPE) cell spaces.^2^ Recent histopathologic studies have observed the CC dysregulation even in early and intermediate AMD promoting angiogenesis and continual vascular remodeling.^3^^–^^5^

Although the pathogenesis of MNV is not completely understood, the mechanism of angiogenesis is characterized by the upregulation of vascular endothelial growth factor (VEGF), secreted by the RPE.^6^ Currently, intravitreal injection of anti-VEGF agents is the most effective therapy to reduce fluid exudation from MNV. Ranibizumab (Lucentis; Genentech, Inc., South San Francisco, CA) and Aflibercept (Eylea; Regeneron Pharmaceuticals, Tarrytown, NY; and Bayer AG, Leverkusen, Germany) are widely available to suppress MNV in AMD.^7^^,^^8^ Furthermore, previous papers showed the pharmacologic anti-VEGF impacts even on the choroid, as visualized by optical coherence tomography (OCT).^9^^,^^10^

With the recent advent of OCT angiography (OCTA), the CC has become accessible for quantitative studies,^4^^,^^5^ and several previous studies have investigated the CC and MNV in AMD eyes.^11^^–^^13^ Importantly, Moult et al.^14^ and Alagorie et colleagues^15^ have analyzed the spatial distribution of the CC impairment around MNV by creating MNV rings to quantify flow deficit (FD) characteristics. However, the current literature lacks evidence regarding CC circulation in response to anti-VEGF therapy. Considering this, it would be important to characterize the impact of intravitreal injections on the CC dropout reflecting the choroidal status.

Therefore, our study aimed to study the distribution of CC flow that surrounds and does not include MNV and dark halo (DH) after loading anti-VEGF therapy in neovascular AMD (nAMD) using OCTA.

## Methods

### Study Participants

This retrospective study analyzed 30 eyes of 30 subjects 55 years of age and older with treatment-naïve AMD and type 1 MNV^16^ undergoing a loading dose of anti-VEGF intravitreal injections (3 monthly anti-VEGF injections) at the Department of Basic Medical Sciences, Neuroscience and Sense Organs, University of Bari “Aldo Moro,” Italy, between August 2021 and March 2022. This study observed the tenets of the Declaration of Helsinki and was approved by the institutional review board of each university.

All patients were imaged with the RTVue XR Avanti spectral-domain (SD)-OCT (Optovue, Inc., Fremont, CA). The OCTA acquisition was performed at the following visits: (i) 0 to 3 days before the first injection of the loading phase of aflibercept or ranibizumab (T1), and (ii) 1 month after the third injection within the loading phase (T2). All patients received a complete ophthalmologic examination, which included the measurement of Snellen best-corrected visual acuity (BCVA), intraocular pressure (IOP), and dilated ophthalmoscopy.

The exclusion criteria included: (i) presence of type 2 or type 3 MNV; (ii) presence of significant cataract; (iii) myopia greater than 3.00 diopters; (iv) history of myocardial infarction or cerebrovascular disease within the last 6 months; (v) infection or inflammation of both eyes; (vi) presence of other comorbid retinal and/or macular diseases (e.g. diabetic retinopathy and retinal venous occlusion); (vii) history of anti-VEGF injection or retinal laser therapy in the study eye; and (viii) any optic neuropathy, including glaucoma.

Moreover, images with a strength index less than 40, with significant motion artifact or shadowing effect were excluded from the analysis.

### Imaging Acquisition

En face OCTA 3 × 3 mm volume scans (500 pixels × 500 pixels resolution) were acquired using the RTvue-XR Avanti (Optovue), a spectral-domain OCTA (SD-OCTA) device that uses an A-scan rate of 70,000 scans per second, a light source centered at 840 nm, and a full-width at a half maximum bandwidth of 45 nm. The RTVue-XR Avanti system acquires 2 consecutive OCTA volume scans (horizontal and vertical), each containing 400 A-scans, and then combined to reduce motion artifacts.^17^

**Figure 1.**
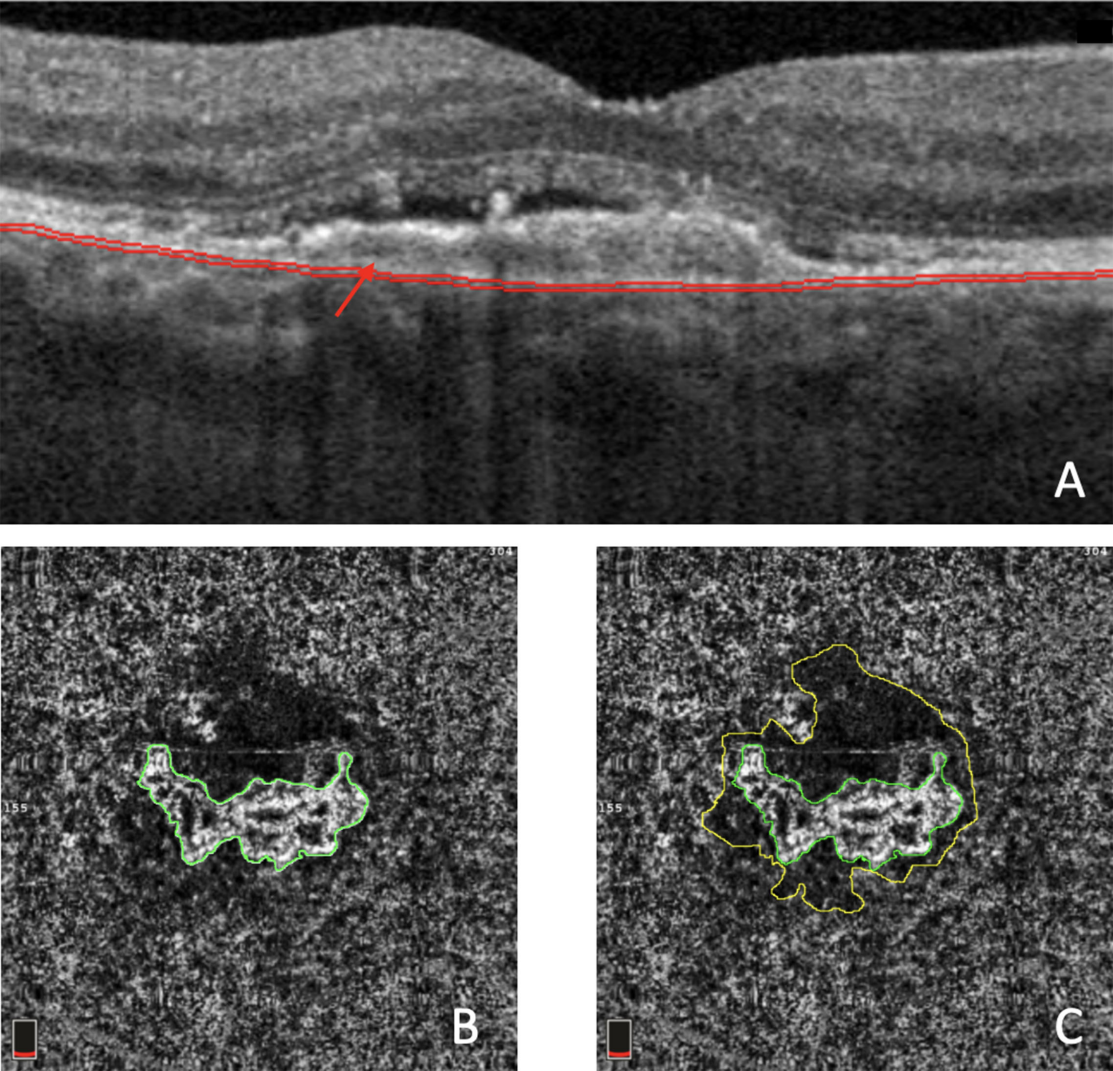
(**A**) Structural OCT B-scan displays the corresponding CC segmentation. CC analysis was performed by analyzing OCTA en face (slab 30 µm thick starting 31 µm posterior to the RPE – Bruch's membrane complex [*red arrow heads*]). (**B**) The 3 × 3 mm en face CC exhibits subfoveal treatment-naïve MNV (highlighted in *green*). (**C**) The perilesional dark halo is *encircled in yellow* and is represented by a low-flow area surrounding the MNV.

The manufacturer's semi-automated segmentation algorithm was used to delimit different retinal layers. The manufacturer's semi-automated segmentation algorithm was used to obtain the CC en face OCTA image (slab 30 µm thick starting 31 µm posterior to the RPE – Bruch's membrane complex). The automatic segmentation was evaluated in every case to ensure accurate referencing of Bruch's membrane and segmentation errors were manually corrected. Manual segmentation was facilitated by the manufacturer's propagation plugin, which can repeat the manually corrected segmentation for several B-scans. CC analysis was performed by analyzing OCTA en face images of the CC slab (10-µm thick, located 21 µm and 31 µm below the anatomic location of Bruch's membrane^18^; Fig. 1). CC areas beneath major superficial retinal vessels were excluded from the analysis to avoid potential shadows or projection artifacts.^19^ Furthermore, to be enrolled in the study, all MNV edges were required to be localized at least 1 mm from the scan edge.

### Image Processing

**Figure 2.**
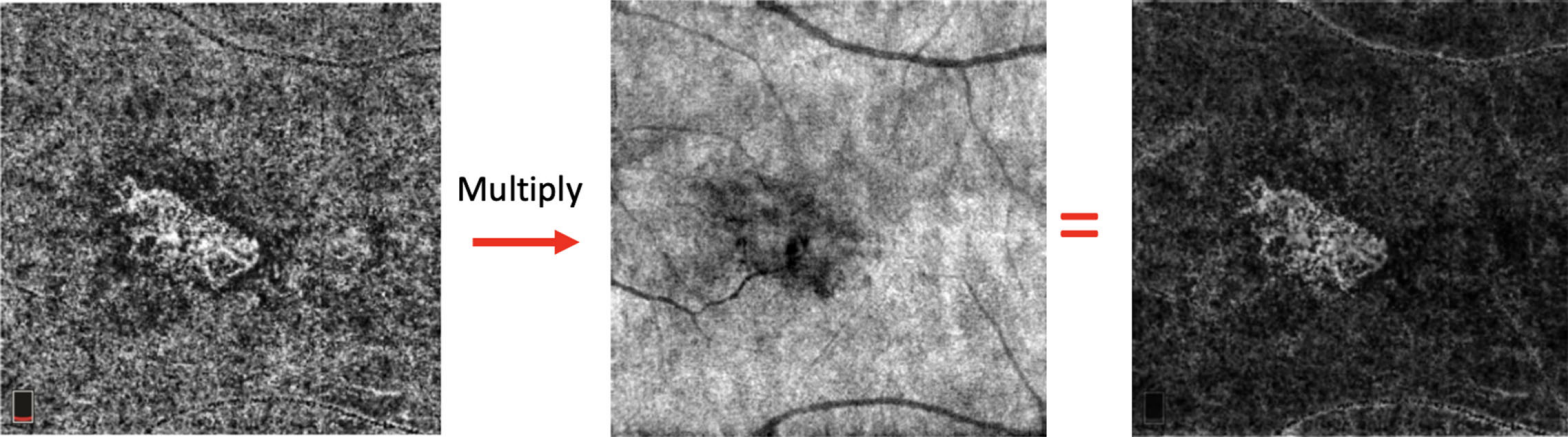
Compensated methodology to remove retinal vessel projection artifacts and to adjust for shadowing artifacts. An inverse transformation process is applied to the en face CC structural to improve the speckle noise of the image. Subsequently, a multiplication process between structural en face CC inverted and the en face CC angiogram is performed.

En face OCTA images of the CC were imported in Fiji ImageJ (software version 2.0.0; National Institute of Health, Bethesda, MD; available at http://rsb.info.nih.gov/ij/index.html) and each CC image was compensated to remove retinal vessel projection artifacts and to adjust for shadowing artifacts using the algorithm proposed by Zhang et al.^20^ (Fig. 2).

**Figure 3.**
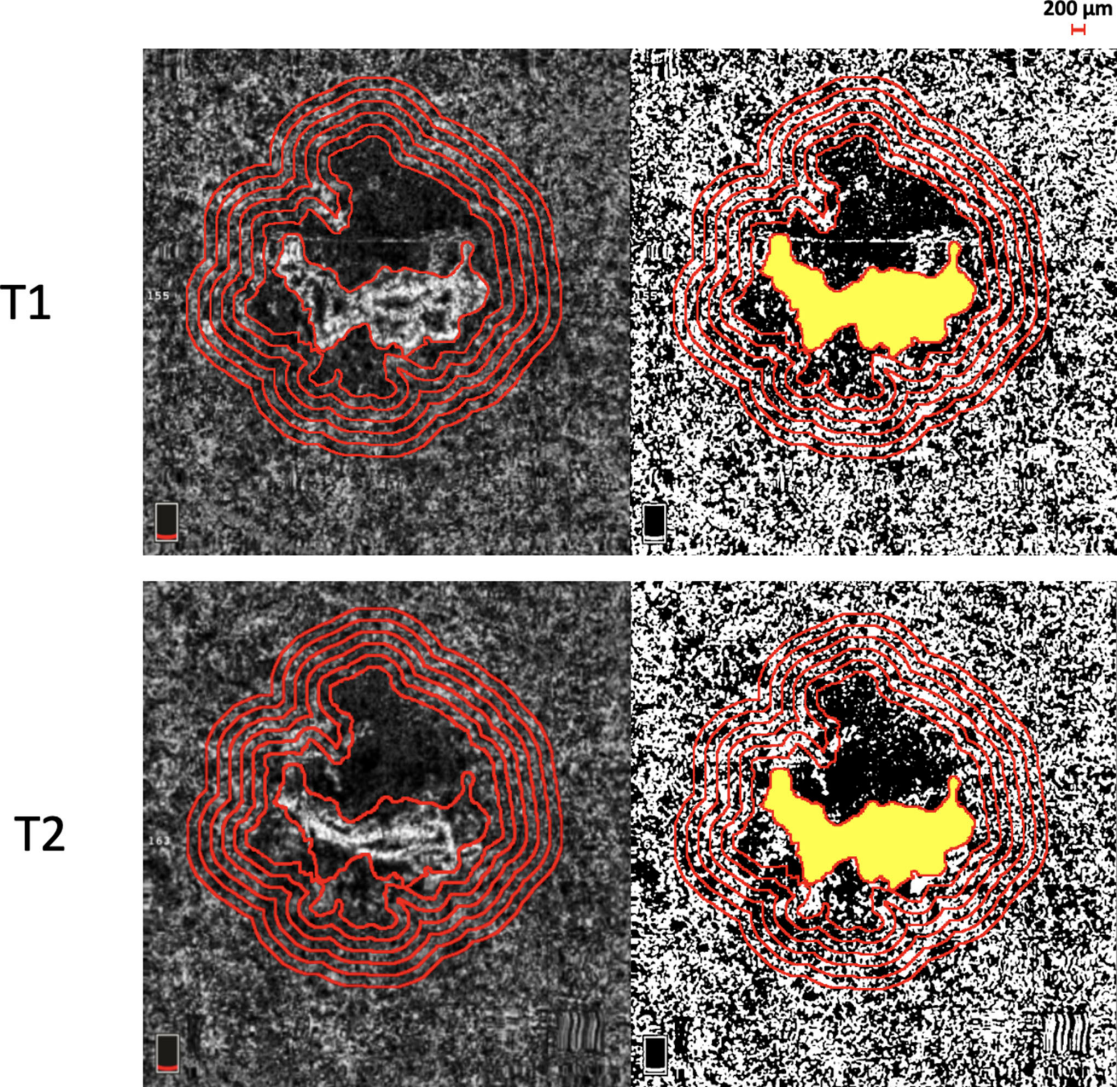
En face CC OCTA images are used to manually delineate the borders of the CNV lesion and the related dark halo. Afterward, 5 progressive 200-µm-wide concentric rings were generated from the edge of the dark halo using the “Distance Map” function in ImageJ, which automatically creates a border that follows the contour of the perilesional halo. Each ring (R1, R2, R3, R4, and R5) was added to the region of interest (ROI) manager for CC flow analysis. The custom configuration (unique for each patient) consisting of these rings was applied to the CC en face at T2 at the same size and position. Then, the resulting CC images were binarized for quantitative measurement of the FD in each ring using the Phansalkar method.

The borders of the CNV lesion and the related dark halo were manually delineated by two masked expert graders (authors PV and MOG) in each en face CC OCTA scan (see Fig. 1). Afterward, following the previous paper,^21^ we spanned a 1000-µm-wide region surrounding the DH around the MNV. Five progressive 200-µm-wide concentric rings were generated from the edge of the DH using the “Distance Map” function in ImageJ, which automatically creates a border that follows the contour of the perilesional halo (Fig. 3). Each ring (R1, R2, R3, R4, and R5) was added to the region of interest (ROI) manager for CC flow analysis. The custom configuration (unique for each patient) consisting of these rings was applied to the CC en face at T2 at the same size and position. Then, the resulting CC images were binarized for quantitative measurement of the FDs in each ring using the Phansalkar method using a radius of 15 pixels, in agreement with previous studies assessing patients with neovascular AMD with the AngioVue device.^22^^–^^24^ (see Fig. 3).

To calculate the CC FD, the “analyze particles” command was used. The CC flow was finally quantified in each ring area (R1, R2, R3, R4, and R5) using the “Analyze Particles” tool provided by ImageJ. In detail, the following metrics were quantified: (i) the FD percentage (FD%) which represents the percentage of flow deficits within the analyzed area (FD%); (ii) the FD average area (FDa) which represents the average size of the FD within the analyzed region; and (iii) the FD number (FDn) which quantifies the number of flow deficits in the ROI.

### Statistics Analysis

All quantitative variables were reported as mean and standard deviation (SD). To detect departures from normality distribution, a Shapiro-Wilk test was performed for all variables. Because CC variables did not display a normal distribution, the nonparametric Friedman test was conducted to compare CC metrics between visits. Statistical calculations were performed using Statistical Package for Social Sciences (version 20.0; SPSS Inc., Chicago, IL). The chosen level of statistical significance was *P* < 0.05.

## Results

### Characteristics of Subjects Included in the Analysis

A total of 30 eyes of 30 Caucasian patients with treatment naïve nAMD were included in the study. Seventeen patients were women and 13 patients were men. The mean age was 73.4 ± 6.5 years (range = 55–85 years). Mean ± SD BCVA was 0.44 ± 0.14 LogMAR at T1 and 0.37  ±  0.18 LogMAR at T2 (*P* = 0.037). In the subjects included in the analysis, the MNV lesion displayed an average mean area of 0.31 ± 0.24 mm^2^ and the related perilesional dark halo measured 2.11 ± 1.96 mm^2^ at T1, and 0.23 ± 0.19 mm^2^ and the related perilesional dark halo measured 1.87 ± 1.34 mm^2^ at T2. The two-way mixed-effects intraclass correlation coefficient was 0.94 (95% confidence interval [CI] = 0.929–0.952) for the MNV area quantification and 0.95 (95% CI = 0.939–0.954) for the dark halo area quantification. The characteristics of the subjects included in the analysis are summarized in Table 1.

**Table 1.** The clinical Characteristics of Subjects Included in the Analysis

	Eyes (*N* = 30)	
Variables	MNV Type		*P* Value
Age, y	73.4 ± 6.5		N/A
Gender, female (%)	17 (56%)		N/A
	T1	T2	
MNV area, mm^2^	0.31 ± 0.24	0.23 ± 0.19	*P* = 0.003
DH area, mm^2^	2.11 ± 1.96	1.87 ± 1.34	*P* = 0.01
BCVA logMAR	0.44 ± 0.14	0.37 ± 0.18	*P* = 0.019

Data are presented as mean ± SD.

MNV, macular neovascularization; DH, dark halo; BCVA, best-corrected visual acuity; T1, before loading anti-VEGF therapy; T2, after loading anti-VEGF therapy; N/A, not applicable; VEGF, vascular endothelium growth factor.

### OCTA Topographical Analysis of the Choriocapillaris

The topographical CC subanalysis showed statistically significant changes in all the subfields analyzed among the different study visits. In detail, all rings showed a progressive FD% reduction at T2 in comparison to T1 values indicating gradual CC reperfusion of the peripheral rings (Table 2). Only the first ring (R1) did not reach statistical significance (52.4 ± 8.3% and 50.3 ± 8.2%, *P* = 0.059; see Table 2).

**Table 2.** OCT Angiography FD % Data and Comparisons (T1 vs T2)

	CC FD (%) T1	CC FD (%) T2	*P* Value
**Ring 1 (R1)**	52.48 ± 8.31	50.46 ± 8.99	0.059
**Ring 2 (R2)**	51.37 ± 8.29	48.30 ± 8.62	0.003
**Ring 3 (R3)**	50.304 ± 8.75	46.73 ± 8.27	0.001
**Ring 4 (R4)**	49.22 ± 9.07	45.77 ± 8.11	0.001
**Ring 5 (R5)**	48.28 ± 9.24	45.10 ± 8.14	0.003

Data are presented as mean ± SD.

FD%, flow deficit percentage; MNV, macular neovascularization; T1, before loading anti-VEGF therapy; T2, after loading anti-VEGF therapy; VEGF, vascular endothelium growth factor.

Likewise, the average size of FD was significantly lower after the loading phase. In particular, we found a progressive contraction of the FD average area in all the rings considered (*P* < 0.05; Table 3). On the other hand, at T2, a significant increase in the FD number of the 5 rings was displayed, as compared to T1 (*P* < 0.05; Table 4).

**Table 3.** OCT Angiography FDa Data and Comparisons (T1 vs T2)

	CC FDa T1	CC FDa T2	*P* Value
Ring 1 (R1)	111.6 ± 63.9	92.4 ± 53.3	0.006
Ring 2 (R2)	84.5 ± 43.1	69.9 ± 36.9	0.004
Ring 3 (R3)	72.9 ± 35.9	58.1 ± 25.8	0.002
Ring 4 (R4)	65.8 ± 32.3	53.4 ± 24.1	0.012
Ring 5 (R5)	60.8 ± 30.1	50.4 ± 23.5	0.014

Data are presented as mean ± SD.

FDa, flow deficit average area; MNV, macular neovascularization; T1, before loading anti-VEGF therapy; T2, after loading anti-VEGF therapy; VEGF, vascular endothelium growth factor.

**Table 4.** OCT Angiography FDn Data and Comparisons (T1 vs T2)

	CC FDn T1	CC FDn T2	*P* Value
Ring 1 (R1)	5333 ± 162.1	6925 ± 232.7	0.025
Ring 2 (R2)	8829 ± 223.2	11638 ± 347.4	0.001
Ring 3 (R3)	12995 ± 295.1	16299 ± 362.1	<0.001
Ring 4 (R4)	17872 ± 379.87	21726 ± 406.9	0.001
Ring 5 (R5)	23223 ± 455.2	27107 ± 437.98	0.001

Data are presented as mean ± SD.

FDn, flow deficit number; MNV, macular neovascularization; T1, before loading anti-VEGF therapy; T2, after loading anti-VEGF therapy; VEGF, vascular endothelium growth factor.

## Discussion

In this OCTA study, we examined the distribution of the CC flow surrounding the dark halo after the loading dose of anti-VEGF therapy in nAMD eyes. We found significant regional differences in the perfusion of the CC after intravitreal injection therapy, which should be taken into account because OCTA-based parameters are used increasingly for nAMD management. At T2, this study found significantly greater CC reperfusion within the 5 concentric rings immediately adjacent to the dark halo, especially in the peripheral rings.

Impairment of the CC surrounding dark halo and CNV has been previously reported. Using postmortem eyes affected by MNV, McLeod et al.^25^ proved that the CC circulation was reduced by 50% in the region surrounding the choroidal neovascularization (CNV), despite a structurally intact overlying RPE. Likewise, in a postmortem study of eight eyes with wet AMD, Biesemeier et al.^26^ highlighted a CC loss preceding the degeneration of the RPE and the overlying retina.

Friedman hypothesized that the etiology of AMD is largely attributable to an impaired choroidal perfusion.

Recent progresses in OCTA confirmed that the CC hypoperfusion is highest in the region surrounding MNV, although signal compensation and image averaging are required to achieve safe quantitative results.^14^^,^^15^^,^^27^ Using a 200 µm ring defined as the “Halo” zone, Treister et al.^28^ demonstrated a significantly greater CC non-perfusion adjacent to all CNV lesions. In a prospective case series of 80 eyes, Coscas et al.^29^ attributed the dark halo presence to the shadowing effect of blood or intraretinal and subretinal fluids, considering it a sign of CNV activity warranting treatment. It is unclear whether the dark halo represents true CC ischemia or the shadow effect.^30^ For this reason, we investigated the CC flow directly outside of the dark halo, excluding the halo itself.

The present study yielded quantitative OCTA evidence in nAMD after intravitreal injection loading. We demonstrated that the CC perfusion remodels after treatment in the area surrounding the dark halo. In detail, the topographical CC subanalysis showed a significant reduction in CC FDa and CC FD% after the loading phase of treatment. The latter results seem to suggest that a CC reperfusion occurs after anti-VEGF treatment in eyes with type 1 MNV and AMD. Importantly, in terms of CC FD%, only the first ring (R1) did not reach statistical significance. These findings could support the hypothesis that the CC immediately adjacent to the dark halo (R1) still suffers from the high flow of CNV by drawing blood from the surrounding regions. Moreover, the analysis conducted toward the peripheral rings shows that the CC is impaired far outside the PH, suggesting that this pathoanatomy may play a role in disease progression.

In addition to reduction in CC FDa and CC FD%, we found an increase of FD number after the loading phase treatment. Together, these findings suggest that a reduced MNV flow signal after intravitreal therapy is associated with tinier FDs that then split, resulting in a greater total number but lower density of FDs.

All of this circumstantial evidence would suggest that active neovascularization may steal adjacent blood flow from surrounding capillaries, even out of the dark halo. This phenomenon is already well described in the neoplastic angiogenic processes. As described by Pilat et al.^31^ cancer cells need to be within a certain distance of a blood vessel to receive enough oxygen and nutrients to survive and proliferate. Similarly, MNV recruits the surrounding vascular system to grow. Supporting this concept, we demonstrated partial recovery of the CC flow after MNV resolution with intravitreal treatment.

It is important to emphasize that the role of anti-VEGF treatment in promoting vascular reperfusion in chorioretinal disease remains unclear and controversial. Although some reports demonstrated adverse vasoconstrictive effects on retinal perfusion^32^^,^^33^ and flow velocities, studies have provided some evidence on the positive effects of anti-VEGF therapy on retinal perfusion.^34^^,^^35^ Papadopulo et al.^32^ hypothesized that anti-VEGF agents decrease retinal arteriolar diameter, by blocking the production of nitric oxide, and may lead to retinal arteriolar vasoconstriction. Contrariwise, using OCTA, long-term administration of intravitreal anti-VEGF injections has been shown to improve or at least maintain retinal perfusion in patients with retinal vein occlusion and diabetic retinopathy.^35^

Our findings could be important for monitoring response to intravitreal injections of anti-VEGF. Our first results appear to confirm in part those of Rispoli et al.^30^ who suggested that dark halo fluctuation seems to be parallel to MNV evolution after treatment. Combined with the latter study, we observed blood flow sequestering around CNV and dark halo before anti-VEGF treatment, followed by increased CC perfusion when CNV activity decreases after treatment. Using OCTA, this is the first study to perform a topographic CC flow analysis before and after loading anti-VEGF therapy.

Our study has several limitations that should be considered when interpreting our findings. First, our sample size was relatively small, and the study design was cross-sectional. Second, we used SD-OCTA which utilizes shorter wavelength light compared with swept-source OCTA, causing a poor penetration signal when passing through the RPE. Another possible limitation of the study is that the qualitative evaluation of en face OCTA, although conducted by two masked graders, could be affected by the subjective nature of the assessment. On the other hand, also the strengths of our study should be kept in mind. For each OCTA scan, we applied for signal compensation, and automatic and manual segmentation error correction and used validated local threshold strategies for FD analysis to maximize reliability.

In conclusion, our study reports topographical CC reperfusion after loading anti-VEGF therapy. We demonstrated the presence and severity of CC FDs around CNV and the dark halo before treatment, followed by a progressive recovery of CC flow after intravitreal therapy. These results yield evidence that the CC layer might play a primary role not only in the development and progression of MNV but also in assessing response to intravitreal injections of anti-VEGF. Furthermore, these results need to be interpreted with caution until further studies are conducted with use of SS-OCT, alternative methods of CC FD assessment, and longer follow-up periods. In the future, larger studies are needed to support our preliminary results.
